# Combined novel homozygous variants in both *SGPL1* and STAT*1* presenting with severe combined immune deficiency: case report and literature review

**DOI:** 10.3389/fimmu.2023.1186575

**Published:** 2023-06-12

**Authors:** Adriel Roa-Bautista, Mahreen Sohail, Emma Wakeling, Kimberly C. Gilmour, Mark Davis, Anthony Gait, Giovanna Lucchini, David Cox, Reem Elfeky, Maaike Kusters

**Affiliations:** ^1^ Paediatric Immunology Department, Great Ormond Street Hospital for Children National Health Service (NHS) Foundation Trust, London, United Kingdom; ^2^ Immunology Unit, Marqués De Valdecilla University Hospital, Santander, Spain; ^3^ North East Thames Regional Genetic Service, Great Ormond Street Hospital for Children National Health Service (NHS) Foundation Trust, London, United Kingdom; ^4^ Great Ormond Street (GOS) Hospital for Children National Health Service (NHS) Foundation Trust, University College London Great Ormond Street (GOS) Institute of Child Health, and National Institute fot Health and Care Research (NIHR), Great Ormond Street Hospital (GOSH), Biomedical Research Centre (BRC), London, United Kingdom

**Keywords:** case report, ichthyosis, lymphopenia, steroid-resistant nephrotic syndrome (SRNS), SGPL1 gene mutation, STAT1

## Abstract

**Background:**

Sphingosine phosphate lyase insufficiency syndrome (SPLIS) is associated with biallelic variants in *SGPL1*, comprising a multisystemic disease characterized by steroid resistant nephrotic syndrome, primary adrenal insufficiency, neurological problems, skin abnormalities and immunodeficiency in described cases. Signal transducer and activator of transcription 1 (STAT1) plays an important role in orchestrating an appropriate immune response through JAK-STAT pathway. Biallelic *STAT1* loss of function (LOF) variants lead to STAT1 deficiency with a severe phenotype of immunodeficiency with increased frequency of infections and poor outcome if untreated.

**Case presentation:**

We report novel homozygous SGPL*1* and *STAT1* variants in a newborn of Gambian ethnicity with clinical features of SPLIS and severe combined immunodeficiency. The patient presented early in life with nephrotic syndrome, severe respiratory infection requiring ventilation, ichthyosis, and hearing loss, with T-cell lymphopenia. The combination of these two conditions led to severe combined immunodeficiency with inability to clear respiratory tract infections of viral, fungal, and bacterial nature, as well as severe nephrotic syndrome. The child sadly died at 6 weeks of age despite targeted treatments.

**Conclusion:**

We report the finding of two novel, homozygous variants in *SGPL1* and *STAT1* in a patient with a severe clinical phenotype and fatal outcome early in life. This case highlights the importance of completing the primary immunodeficiency genetic panel in full to avoid missing a second diagnosis in other patients presenting with similar severe clinical phenotype early in life. For SPLIS no curative treatment is available and more research is needed to investigate different treatment modalities. Hematopoietic stem cell transplantation (HSCT) shows promising results in patients with autosomal recessive STAT1 deficiency. For this patient’s family, identification of the dual diagnosis has important implications for future family planning. In addition, future siblings with the familial *STAT1* variant can be offered curative treatment with HSCT.

## 1 Introduction

Sphingosine phosphate lyase insufficiency syndrome (SPLIS) is a multisystemic condition associated with biallelic pathogenic variants in *SGPL1* (OMIM no. 603729) (1). This recently described syndrome (2017) comprises a broad phenotype, featuring in most patients a steroid-resistant nephrotic syndrome, endocrine, dermatological, and neurological system involvement (2–4). In addition, lymphopenia has been previously described (5) but the significance in the clinical presentation of patients still needs to be further elucidated (6).

Sphingosine-1-phosphate lyase (SPL) is an intracellular enzyme involved in the final step for sphingolipid degradation (7). Specifically, SPL catalyses the cleavage of sphingosine-1-phosphate (S1P) resulting in the formation of other essential biomolecules (5) needed to mediate biological activities such as cell migration, survival, and proliferation (7). Furthermore, S1P signaling regulates T cell egress from the thymic tissue and other peripheral lymphoid organs (8) which could explain the lymphopenia in some patients.

Signal transducer and activator of transcription 1 (STAT1) is an important member of the STAT family, playing a role in cell growth, differentiation, proliferation, metabolism, and apoptosis through the JAK-STAT pathway (9). Biallelic *STAT1* loss of function (LOF) variants result in a severe phenotype of immune deficiency with increased susceptibility of patients to bacterial, viral, and mycobacterial disease (OMIM no. 600555) (10).

Herein, we describe a newborn with novel homozygous variants in both *SGPL1* and STAT*1* presenting with severe combined immune deficiency and additional clinical features.

## 2 Case description

### 2.1 Clinical presentation

A female baby was born at 39 + 6 weeks gestation to healthy, Gambian parents. Parents were not known to be related but originated from the same tribe. Parents had three other healthy children. There was a previous history of one spontaneous first trimester miscarriage.

During the prenatal period, the patient was noted by ultrasound to have right-sided chest effusion that was treated with pleural-amniotic intrauterine shunt insertion at 36 weeks gestation. The shunt spontaneously came out on the second day of life. The patient was born by spontaneous vaginal delivery and intubated at 8 minutes of life due to irregular breathing with increased oxygen requirements. She was admitted to the Neonatal Intensive Care Unit for 8 days due to respiratory distress and pleural effusion. The diagnosis of sepsis was raised due to positive maternal colonization with Group B Streptococcus. Intravenous antibiotics were commenced, but subsequently discontinued when blood culture results came back as negative.

She failed her neonatal hearing screen bilaterally. She was also noted to have dry and scaly skin (no ectropion or eclabium) raising the possibility of a congenital ichthyosis by the local dermatology team. The patient was discharged on day 8 of life and re-admitted on day 13 with cough, coryza, reduced feeding, increased work of breathing and tachypnoea with no fever. Continuous positive airway pressure was started but intubation was required due to respiratory deterioration. Empiric antibiotic and antiviral therapy (Piperacillin-Tazobactam, Amikacin and Acyclovir) were started.

**Figure 1:**
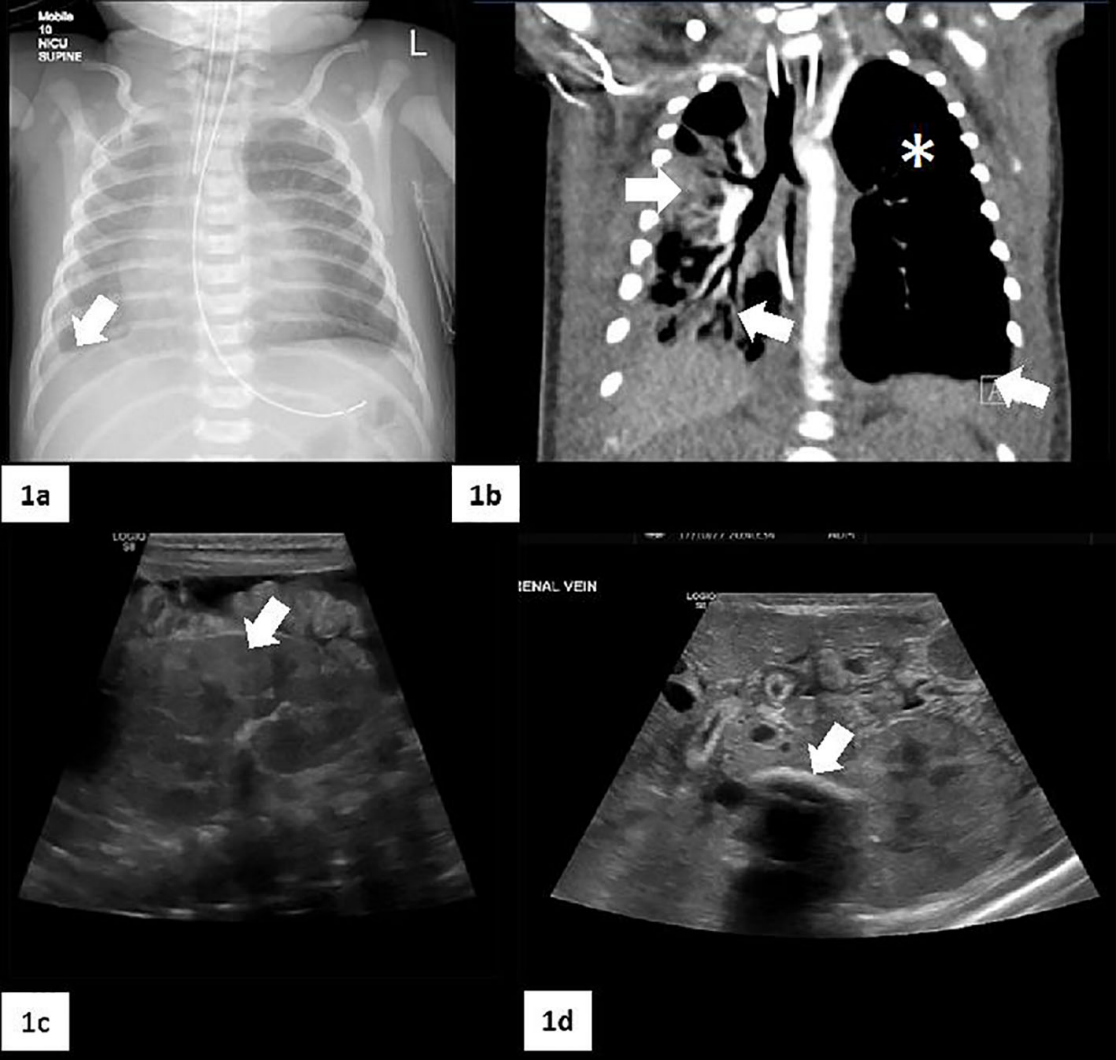
Radiological imaging. **(A)** Chest X-Ray that shows a moderate right pleural fluid collection (white arrow) accompanied by a volume loss in the right lung. **(B)** CT scan: small right lung with a predominantly central/perihilar consolidation with air bronchograms (two white arrows). The left lung is slightly hyperexpanded, with no interstitial changes and small areas of atelectasis (white asterisk). There are small bilateral pleural effusions (white arrow). **(C)** Marked abnormality of the kidneys with cortical echogenicity (white arrow). **(D)** Shows a calcified left renal vein thrombosis (white arrow). Free fluid in the peritoneum with some internal strands suggestive of chronicity.

ECG showed ongoing sinus bradycardia; echocardiogram was normal. Full sepsis screen was carried out. Bronchoalveolar lavage (BAL) and nasopharyngeal aspirate were positive for enterovirus, rhinovirus and Serratia marcescens (resistant to Piperacillin-Tazobactam). Blood and urine cultures showed no growth. Cerebrospinal fluid (CSF) examination was normal with normal cell count, no organisms on CSF culture. CT chest showed a hypoplastic right lung and mild right sided pleural effusion (**Figure 1**).

The patient was edematous with significant and persistent hypoalbuminemia despite albumin infusion. In the absence of diarrhea and with a normal fecal alpha-1-antitrypsin, protein losing enteropathy was excluded. Low levels of albumin <20 (34-42 g/L) with raised urine/albumin ratio confirmed nephrotic syndrome. A left calcified renal vein thrombus was detected on renal ultrasound thus low molecular weight heparin was started. At 21 days of life, the patient’s clinical condition deteriorated requiring high frequency oscillatory ventilation.

Full blood counts on admission showed marked lymphopenia; total lymphocytic count of 750 cells/ul, CD3 of 310 cells/ul, CD19 130 cells/ul, CD4 280 cells/ul, CD8 20 cells/ul and CD16 260 cells/ul. She had low naïve CD4 110 cells/ul with low recent thymic emigrants (9.3%) and reduced T cell receptor excision circles (TRECS) of 196 x10*6/L (0-1160 μl). Results on new born screening for SCID was back on day 13^th^ of life and showed zero TRECS on the Gurthie card. She had low levels of IgG 2.65 (3.90-13.00G/L), absent IgA, less than 0.07 (0.02-0.15 G/L) and normal IgM 0.25 (0.08-0.4 G/L) and normal expression of MHC class I protein (98%). The constellation of ichthyosis, hearing loss, nephrotic syndrome, and lymphopenia, raised the possibility of a causative *SGPL1* variant. Next generation sequencing with a primary immune deficiency (PID) gene panel, including *SGPL1*, was initiated.

Endocrine work-up showed normal adrenal function: ACTH 17 (10-50ng/L), aldosterone 1,060 pmol/L (<1,000 pmol/L), cortisol 381 (110-560nmol/L) and no adrenal calcification. However, thyroid function tests were abnormal, with low T4 7.0 (9.0-19.6 pmo/L), low T3 3.0 (5.1-10.0 pmol/L), and high thyroid-stimulating hormone (TSH) 12.6 (<6.0 mU/L) with no evidence of anti-thyroid peroxidase antibody (TPO) or thyroid stimulating hormone receptor antibodies (TRab) pointing to the presence of hypothyroidism for which Levothyroxine was started.

At 24 days of life, the clinical condition of the patient continued to deteriorate with ongoing respiratory acidosis and raised C-Reactive protein (195mg/L). A positive BAL for Candida parapsilosis was obtained, and Caspofungin was started.

**Figure 2:**
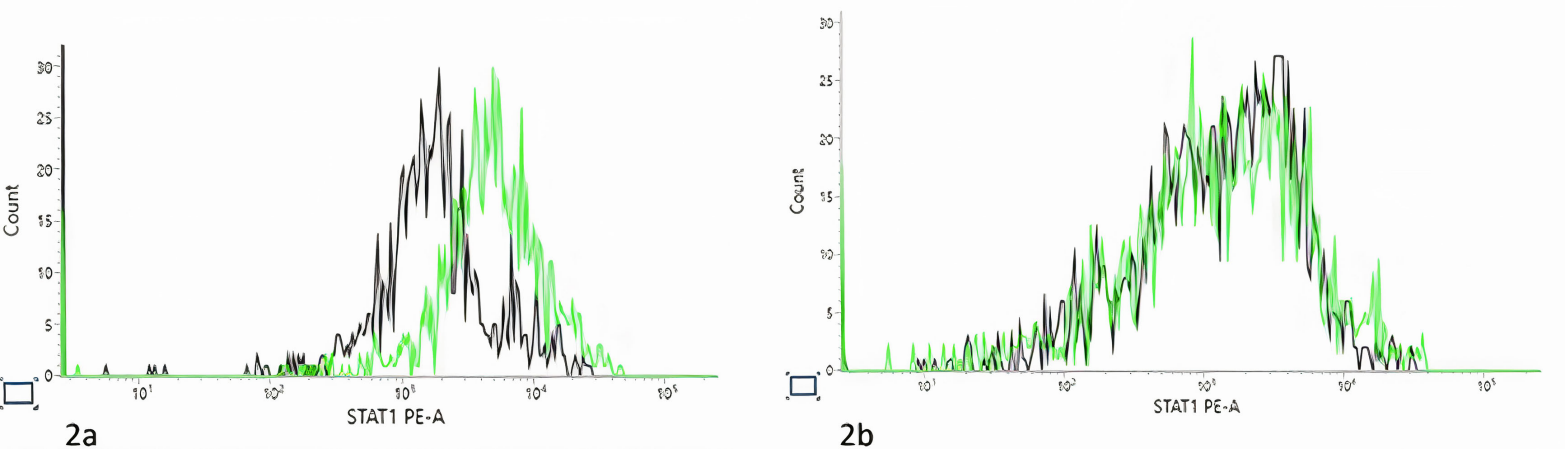
Signal transducer and activator of transcription 1 (STAT1) phosphorylation assay. Black line, unstimulated fibroblast. Green line, post stimulation with IFNα. **(A)**. Control STAT1 Phosphorylated Tyrosine Measurement. Black line, number of acquired events= 1416. Median fluorescence intensity (MFI)= 2639; Green line, number of acquired events= 1429, MFI= 3146. **(B)**. Patient STAT1 Phosphorylated Tyrosine Measurement. Black line, number of acquired events= 941, MFI= 2766; Green line, number of acquired events= 917, MFI= 6858). As there patient fibroblast grow poorly, the experiment was preformed one time, and as many cells as possible were acquired during the experiment.

Next generation sequencing showed a homozygous missense variant in *SGPL1*: c.1027G>C p. (Gly343Arg). This variant was previously unreported but in silico was predicted to have a deleterious effect. In addition, the patient was found to be homozygous for a *STAT1* variant: c.945-12G>A. This variant was also previously unreported. Functional assays in fibroblast cell lines of the patient showed low STAT1 phosphorylation in comparison with healthy control (**Figure 2**). Parents were found to be heterozygous for both *SGPL1* and *STAT1* variants.

**Figure 3:**
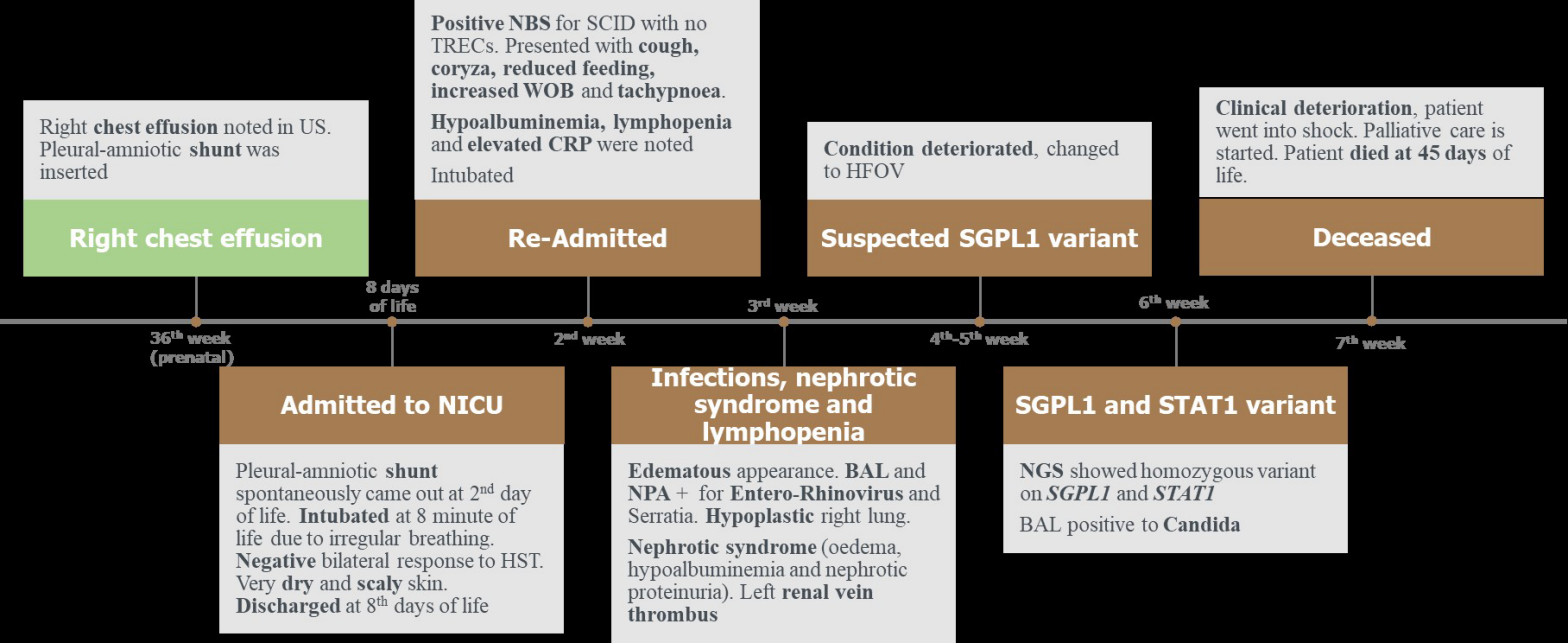
Timeline of the clinical presentation of the patient. BAL, bronchoalveolar lavage. CRP, C-reactive protein. HST, hearing screening test. HFOV, high frequency oscillatory ventilation. NBS, newborn screening. NPA, nasopharyngeal aspiration. NICU, neonatal intensive care unit. NGS, next generation sequencing. SCID, severe combined immunodeficiency. *STAT1*, signal transducer and activator of transcription. *SGPL1*, sphingosine 1-phosphate lyase. US, ultrasound. WOB, work of breathing.

Subsequently, the patient went into shock, inotropes were added, and bicarbonate correction was given due to increased acidosis. The patient’s clinical situation did not improve despite multiple therapeutic interventions and sadly the patient died at 45 days of life (**Figure 3**).

### 2.2 Timeline

Data shown in **Figure 3**.

### 2.3 Genetic and functional assays

#### 2.3.1 Genetic testing

Analysis of a virtual panel of 255 genes associated with PID was undertaken on whole exome data (TWIST Human Core Exome and Illumina sequencing) generated from DNA extracted from venous blood. Variant calling, annotation and filtering was carried out by a custom in-house pipeline and variant interpretation conducted in line with the ACMG/AMP guidelines (11) and ACGS best practice guidelines for variant classification in rare disease (12). Two homozygous variants of interest were identified in the *SGPL1* and *STAT1* genes. Confirmation and parental testing were carried out by targeted Sanger sequencing.

#### 2.3.2 Variant interpretation

*SGPL1* had been raised as a gene of interest by both the patient’s clinical genetics consultant and immunology consultant due to the patient’s pattern of clinical features. A homozygous missense variant c.1027G>C p. (Gly343Arg) (NM_003901.3) in *SGPL1* was identified and predicted to be deleterious by in silico software. The variant was absent from the gnomAD population database (13). The variant was discussed at a multidisciplinary meeting where it was agreed that the specificity of the patient’s clinical features was sufficient to report the variant as likely to be pathogenic with the following ACMG criteria: PM2 moderate, PP4 moderate, PP3 supporting and PM3 supporting.

An additional homozygous intronic variant c.945-12G>A (NM_007315.3) was identified in the *STAT1* gene. The variant was predicted to have a deleterious effect on splicing by in silico tools and was absent from the gnomAD population database. Subsequent functional analysis of patient fibroblasts showed abnormal STAT1 phosphorylation. The variant was classified as likely to be pathogenic with the following ACMG criteria: PM2 moderate, PP4 moderate, PP3 supporting and PM3 supporting.

#### 2.3.3 Functional assays

To detect STAT1 phosphorylation, patient fibroblasts were either left unstimulated or stimulated with 105 units/ml of IFNα (Stratech Scientific Limited). Cells were fixed, washed and permeabilized before adding 5μl anti-STAT1 phosphorylated tyrosine antibody (BD Bioscience) as previously described (14). Ten thousand fibroblasts were acquired and analyzed (FACsLyrics, BD Biosciences). The percentage change in phosphorylated cells was calculated by subtracting the percentage of unstimulated cells from that of the stimulated cells.

## 3 Discussion

We report two novel likely pathogenic variants in *SGPL1* and *STAT1* that, in combination, led to increased propensity for opportunistic infections in a patient with additional features of hearing loss, nephrotic syndrome and ichthyosis.

In 2017, several groups reported biallelic *SGPL1* variants in association with SPLIS - a multi-systemic disease including primary adrenal insufficiency, ichthyosis, and steroid resistant nephrotic syndrome (2, 15, 16). We did a literature search and to date there are 59 reported cases with SPLIS including our patient (**Table 1**; **Supplementary Table 1**) with median age of presentation of 0.7 months (range: prenatal to 15 years) and a wide spectrum of clinical symptoms. Children presenting early in life had a more severe clinical presentation with primary adrenal insufficiency or steroid-resistant nephrotic syndrome being seen in the early postnatal period. Overall mortality rate is high, approaching 47% (17).

**Table 1:** Summary of the reported cases of SPLIS (59 cases).

Age of onset. Median (range)	0.7 months (Prenatal to 15 years)	
	No of patients (n=59)	Percentages
Sex		
Female	27	45.8
Male	29	49.2
Not Available	2	3.4
Parental consanguinity		
Yes	34	57.6
No	10	16.9
Not Available	15	25.4
Most frequent mutations		
C.665G>A	11	18.6
c.1018C>T	5	8.5
c.1037G>6	5	8.5
Endocrine 47/59 (79.7%)		
Primary adrenal insufficiency	39	66.1
Hypothyroidism	17	28.8
Adrenal Calcifications	16	27.1
Microphallus and cryptorchidism	12	20.3
Hyperparathyroidism	2	3.4
Renal 47/59 (79.7%)		
Steroid-Resistant Nephrotic syndrome	30	50.9
Glomerulopathy	28	47.5
*FSGS*	*23*	*82.1*
*IgM nephropathy*	*1*	*3.6*
*MesPGN*	*2*	*7.1*
*DMS*	*2*	*7.1*
End stage renal disease	18	30.5
Oedema	10	16.9
Haematuria	1	1.7
Neurology 32/54 (53.2%)		
Seizures	12	20.3
Developmental delay	11	18.6
Hearing impairment	10	16.9
Microcephaly	7	11.9
Peripheral/axonal neuropathy	7	11.9
Developmental regression	6	10.2
Generalized hypotonia	5	8.5
Ataxia	4	6.8
Chorea	1	1.7
Macrocephaly	1	1.7
Immunology 30/59 (50.8%)		
Lymphopenia	23	38.9
History of infections	12	20.3
Hypogammaglobulinemia	5	8.5
Autoimmunity	2	3.4
Skin 26/59 (44.1%)		
Ichthyosis	17	28.8
Hyperpigmentation	13	22.0
Scaly skin	1	1.7
Calcinosis cutis	1	1.7
Metabolic 15/58 (25.4%)		
Hypoalbuminemia	7	11.9
Hypoglycaemia	7	11.9
Hypertriglyceridemia	4	6.9
Ophthalmology 8/58 (13.5%)		
Ptosis	4	6.9
Strabismus	4	6.9
Outcome		
Alive	26	44.1
Deceased	28	47.5
Not Available	4	6.8

FSGS, focal segmental glomerulosclerosis. MesPGN, mesangial proliferative glomerulonephritis. DMS, diffuse mesangial sclerosis.

There is no clear genotype/phenotype correlation in terms of morbidity and mortality. Various variants have been described, with three recurrent variants accounting for one third of reported cases so far.

There is a wide spectrum of presentation with multiple system involvement. The main affected systems are endocrine (80%), renal (80%) and neurological features (53%) in reported cases. Primary adrenal insufficiency is the most common endocrine manifestation (66%). Steroid-resistant nephrotic syndrome accounted for the most frequent renal manifestation (51%). Neurological dysfunction affecting both central and peripheral nervous system has been described (53%), including hearing impairment, seizures, developmental delay, and peripheral axonal neuropathy. Ichthyosis is the most frequently reported skin manifestation.

Immunological abnormalities reported include lymphopenia (39%), hypogammaglobulinemia (8%), with a history of infections reported in 20% (**Table 1**). Not all reported cases underwent an immunological workup. Specific patterns of infections (viral/fungal/bacterial) are not described in detail in most cases, and limited data is available regarding their contribution to mortality. Mortality is mainly caused by respiratory failure secondary to steroid resistant nephrotic syndrome, without a specific emphasis placed on whether immunodeficiency and presence of infections could have contributed to increased risk of mortality. Further studies still are needed to define the cause and role of the disrupted immune system in this condition, and to establish if there is a genotype-phenotype correlation.

Our patient presented early in life with respiratory infections and nephrotic syndrome, representing the more severe end of the spectrum of cases described. Lymphopenia and hypogammaglobulinemia could both be caused by primary immunodeficiency due to lack of T cells leaving the thymus mimicking a severe combined immunodeficiency (SCID) phenotype. In addition, secondary immunodeficiency due to protein losses in the context of severe nephrotic syndrome as well as protein and cellular losses due to pleural effusions are important contributing factors as well for the severe lymphopenia present in our patient. Both primary and secondary immunodeficiency lead to severe T-cell lymphopenia and are likely to be picked up through newborn screening for SCID. Interestingly, of the other patients with SPLIS presenting with severe clinical phenotypes early in life, four cases were picked up during newborn screening for SCID due to low TRECs at birth (6, 18).

Our patient suffered from opportunistic infections including Serratia marcescens, enterovirus and pulmonary candida infection and was unable to clear these infections despite targeted antimicrobial treatments. This prompted the medical team to analyze the full PID gene panel despite an early diagnosis of SPLIS.

In addition to the homozygous *SGPL1* variant, an additional homozygous *STAT1* variant was found in our patient, raising the possibility of dual diagnosis with overlapping presentations.

STAT1 is one of the most important members of the STAT family, playing a critical role in regulating cell growth, differentiation, proliferation, metabolism, and apoptosis through the JAK-STAT pathway (19). STAT1 is critical for cellular response to IFNA/IFNB (type 1 interferon) and IFNG (type III interferon). STAT 1 LOF variants can be associated with both autosomal dominant and autosomal recessive immunodeficiency. Heterozygous *STAT1* LOF variants selectively affect the IFNG pathway and cause impairment of mycobacterial but not viral immunity (20). Biallelic LOF variants were first described in 2003 in patients showing features of combined immunodeficiency with increased predisposition to both mycobacteria and viral infections (21, 22). Patients with STAT1 deficiency due to biallelic LOF variants have impaired response to both IFNA/IFNB and IFNG.

Autosomal recessive STAT1 deficiency can be partial or complete (10). The phenotype is similar, however, patients with a partial deficiency present with a milder clinical course (23, 24). The largest series to date of complete STAT1 deficiency includes 24 patients (from 10 families) with 17 different variants and high mortality rate of 65%, mainly due to significant infections (19). In line with the previous described cases, our patient with complete STAT1 deficiency suffered from life threatening opportunistic viral (enterovirus), bacterial (Serratia) and fungal (candida) infections. She did not receive BCG vaccine.

At present, there is no curative therapy for SPLIS, but multiple therapeutic approaches have been implemented, such as renal transplantation, hormone replacement, and exogenous administration of pyridoxine (a co-factor for S1P lyase). Other potential treatments are under evaluation, in particular, enzyme replacement, gene therapy and CRISPR gene editing (1). Patients with complete STAT1 deficiency have poor prognosis with disseminated infections causing death in the first year of life (10, 25). HSCT has been considered as a therapeutic approach to these patients, and promising results have been published in the literature (25, 26).

In conclusion, we report a patient with homozygous pathogenic variants in both *SGPL1* and *STAT1*, leading to dual diagnosis of SPLIS and complete STAT1 deficiency. Both conditions can cause severe combined immunodeficiency and increased susceptibility to infections with poor outcome, as was sadly the case in our patient.

The finding of two homozygous variants highlights the importance of completing broad PID gene panel analysis. This is particularly the case in patients with complex clinical phenotypes, atypical findings and/or possible or known consanguinity.

The dual diagnosis has important genetic counselling implications. There is a separate recurrence risk of 25% for each condition in future pregnancies. Options for future pregnancies, including prenatal and preimplantation diagnosis can be discussed with the family. Carrier testing for both conditions can also be offered to other family members to inform their reproductive choices. Finally, for future children complete STAT1 deficiency is potentially curable with HSCT.

## Data availability statement

The original contributions presented in the study are included in the article/**Supplementary Material**. Further inquiries can be directed to the corresponding author.

## Ethics statement

Written informed consent was obtained from the minor(s)’ legal guardian/next of kin for the publication of any potentially identifiable images or data included in this article.

## Author contributions

AR-B, EW, KG, AG, DC, RE and MK: Conceptualization, Visualization, Writing - review & editing. AR-B, MS, MD, GL, KG, AG, RE and MK: Data curation, Methods, Investigation. AR-B, RE and MK: Writing - original draft, Writing - review & editing. KC, DM and AG: Methods, Software. EW, KG, RE and MK: Supervision, Writing - review & editing. All authors contributed to the article and approved the submitted version.
