# Supplementary material for: Combined novel homozygous variants in both SGPL1 and STAT1 presenting with severe combined immune deficiency: case report and literature review
Source: Front Immunol. 2023 Jun 12;14:1186575. doi: 10.3389/fimmu.2023.1186575 (PMC10291229; doi:10.3389/fimmu.2023.1186575)
Supplement: Supplementary file 1 [file Table_1.docx]

**Supplementary Table 1:** Summary of mutations and outcome of the reported cases with SPLIS (59 cases)

| Reference | Nucleotide change | Amino acids change | Outcome | Other relevant data |
| --- | --- | --- | --- | --- |
| (Tastemel Ozturk *et al.*, 2023) | c.1018>T | p.Arg340Tpr | Alive | Kidney transplant (5.7y). Tetra ventricular hydrocephalus. Left cardiac ventricular hypertrophy. |
| (Tastemel Ozturk *et al.*, 2023) | c.1018>T | p.Arg340Trp | Alive | Hypertension. Humoral immunodeficiency. Thin corpus callosum and increased depth and width of the cerebral sulcus |
| (Linhares *et al.*, 2018) | c.1018C>T | p.Arg340Trp | Deceased (3.5y) | High levels of cholesterol. Pericardial effusion and cardiomegaly. Multiple episodes of high blood pressure |
| (Pezzuti *et al.*, 2014) | c.1018C>T | p.Arg340Trp | Deceased (1.5y) | Patients is the older brother of subject 0628. No genetic study is available. We assume that the genetic mutation is the same? |
| (Seven Menevse *et al.*, 2022) | c.1018C>T | p.R340W) | NA |  |
| (Lovric *et al.*, 2017) | c.1037G>T | p.Ser346IIe | Deceased (3m) | Hypocalcaemia |
| (Lovric *et al.*, 2017) | c.1037G>T | p.Ser346IIe | Deceased (5m) | Prenatal oedema/hydrops. Muscular hypotonia. Intestinal malformation. Facial dysmorphism. Hypocalcaemia |
| (Lovric *et al.*, 2017) | c.1037G>T | p.Ser346IIe | Deceased (1m) | Muscular hypotonia. Foetal hydrops |
| (Lovric *et al.*, 2017) | c.1037G>T | p.Ser346IIe | Deceased (36w) | Foetal hydrops |
| (Lovric *et al.*, 2017) | c.1037G>T | p.Ser346IIe | Deceased (NA) | Foetal demise with hydrops fetalis |
| (Maharaj *et al.*, 2022) | c.1049A>G | p.D35G | Alive | Ovarian calcification |
| (Zhao *et al.*, 2020) | c.1077del;c.1058A>G | p.G360Afs*49;p.K353R | Deceased (2m) | Low TRECs. |
| (Tastemel Ozturk *et al.*, 2023) | c.1079G>T | p.Gly360Val | Deceased (7m) | Hypertension. |
| (Zhao *et al.*, 2020) | c.1079G>T | p.G360V | Deceased (4.5y) |  |
| (Bamborschke *et al.*, 2018) | c.1233delC | p.Phe411Leufs*56 | Deceased (6w) | Foetal hydrops. Dysmorphic facial features. Cortical atrophy. Cerebellar hypoplasia. |
| (Lovric *et al.*, 2017) | c.1247A>G | p.Tyr416Cys | Alive | Anaemia. Failure to thrive. Family history of immune disease related dead. Dilated cardiomyopathy |
| (Janecke *et al.*, 2017) | c.1513C>T | p.Arg505* | Deceased (3m) |  |
| (Janecke *et al.*, 2017) | c.1513C>T | p.Arg505* | Deceased (7w) |  |
| (Prasad *et al.*, 2017) | c.1633_1635delTTC | p.F545del | Alive | Failure to thrive. Vomiting. Salt and pepper retinopathy. Low CD3+, CD4+ naïve but normal proliferation capacity. |
| (Tastemel Ozturk *et al.*, 2023) | c.1635_1637delCTT | p.Phe545del | Deceased (8.6y) | Night blindness. Cerebral and cerebellar atrophy |
| (Prasad *et al.*, 2017) | c.261+1G>A | p.565rFS*6 | Alive | Cryptorchidism and bilateral micro-orchidism. Hypercholesterolemia. Anaemia. Neutropenia. Enlargement of adrenal glands. Kidney transplant. |
| (Prasad *et al.*, 2017) | c.261+1G>A | p.565rFS*6 | Alive | Bilateral cataracts. Anaemia. Raised cholesterol |
| (Lovric *et al.*, 2017) | c.395A>G/c.832delA | p.Glu132Gly. Exon 5 skipping/p.Arg278Glysfs*17 | Alive |  |
| (Lovric *et al.*, 2017) | c.395A>G/c.832delA | p.Glu132Gly. Exon 5 skipping/p.Arg278Glysfs*17 | Alive |  |
| (Zhao *et al.*, 2020) | c.44A>G | p.Y15C | Alive | Low CD3. Family history of similar neurological problems. |
| (Maharaj *et al.*, 2020) | c.511A>G | p.N171D | Deceased (9m) | Failure to thrive. |
| (Seven Menevse *et al.*, 2022) | c.518T>A | p.L173Q | NA |  |
| (Atkinson *et al.*, 2017) | c.55T>C;c.1082C>G | p.Ile184Thr;p.Ser361 | Alive | Mild scoliosis. Charcot-Marie-Tooth neuropathy |
| (Atkinson *et al.*, 2017) | c.55T>C;c.1082C>G | p.Ile184Thr;p.Ser361 | Alive | Charcot-Marie-Tooth neuropathy |
| (Mathew *et al.*, 2022b, 2022a) | c.605C>T | p.Ser202Leu | Deceased (7d) | Prenatal polyhydramnios. Global hypotonia. |
| (Zhao *et al.*, 2020) | c.605C>T, c.1247A>G | p.S202L, p.Y416C | Alive |  |
| (Lovric *et al.*, 2017) | c.605C>T/c.946G>A | p.Ser202Leu/p.Ala316Thr | Alive | Mononeuritis multiple. T/B/NK lymphopenia |
| (Lovric *et al.*, 2017) | c.664C>T | p.Arg222Trp | Deceased (pre-natal) | Foetal demise |
| (Lovric *et al.*, 2017) | c.664C>T | p.Arg222Trp | Deceased (pre-natal) | Foetal demise |
| (Lovric *et al.*, 2017) | c.664C>T | p.Arg222Trp | Deceased (1m) | Immune deficiency (Low levels of T and B). Hypoplasia of corpus callosum. Anaemia. Failure to thrive, capillary leak syndrome |
| (Lovric *et al.*, 2017) | c.664C>T | p.Arg222Trp | Deceased (2m) | Foetal hydrops |
| (Tastemel Ozturk *et al.*, 2023) | c.665G>A | p.Arg222Gln | Deceased (4.4y) | Cerebral volume loss. Left heart ventricle hypertrophy. |
| (Settas *et al.*, 2019) | c.665G>A | p.R222Q | Alive | Limited information available |
| (Zhao *et al.*, 2020) | c.665G>A | p.R222Q | Alive | Dialysis (9y) |
| (Prasad *et al.*, 2017) | c.665G>A | p.R222Q | Alive | Kidney transplant (5y). Siblings with similar clinical history. |
| (Lovric *et al.*, 2017) | c.665G>A | p.Arg222Gln | Alive | Transplant at 7y. *Median and ulnar nerve paralysis |
| (Lovric *et al.*, 2017) | c.665G>A | p.Arg222Gln | Alive |  |
| (Lovric *et al.*, 2017) | c.665G>A | p.Arg222Gln | Deceased (2.9y) |  |
| (Prasad *et al.*, 2017) | c.665G>A | p.R222Q | NA |  |
| (Prasad *et al.*, 2017) | c.665G>A | p.R222Q | NA | Mineralocorticoid deficiency. |
| (Prasad *et al.*, 2017) | c.665G>A | p.R222Q | NA |  |
| (Settas *et al.*, 2019) | c.665G>A | p.R222Q | Alive |  |
| (Tastemel Ozturk *et al.*, 2023) | c.715dupC | p.Gln239fs*8 | Deceased (35d) |  |
| (Lovric *et al.*, 2017) | c.7dup | p.Ser3Lysfs*11 | Alive |  |
| (Lovric *et al.*, 2017) | c.7dup | p.Ser3Lysfs*11 | Alive | Deficiency cellular immunity. Abnormal gait |
| (Lovric *et al.*, 2017) | c.7dup | p.Ser3Lysfs*11 | Alive | Acrocomia, asymmetric skull, moderate scoliosis |
| (Prasad *et al.*, 2017) | c.7dupA | p.S3Kfs*11 | Alive | Kidney transplant (x2) Chronic rejection of the first graft. |
| (Zhao *et al.*, 2020) | c.868-T>C, c.993C>G | p.F290L, p.Y331* | Deceased (7m) | Prenatal polyhydramnios and bilateral adrenal haemorrhages. |
| (Taylor *et al.*, 2019) | c.868T>C;c.993C>G | p.Phe290Leu;p.Tryr331* | Deceased (12m) | Polyhydramnios (prenatally diagnosed). Hyperkeratosis. Neutropenia. Calcification of basal ganglia |
| (Tran *et al.*, 2023) | c.932C>G | p.Pro311Arg | Deceased (19d) | Diagnosed of SCID due to low TRECs. Lymphadenopathies. |
| (Janecke *et al.*, 2017) | c.934delC | p.Leu312Phefs*30 | Alive | Unilateral hydrothorax and generalized skin oedema (Intrauterine) |
| (Tran *et al.*, 2023) | c.946G>A | p.Ala316Thr | Alive | Hypertension. Left ventricular hypertrophy. Dizygotic twin sibling expired at 6y from renal failure. secondary to nephrotic syndrome. Hypertrichosis, xerosis. Short stature |
| (Zhao *et al.*, 2020) | c1566+2T>C;c.854G>A | p.C285Y | Deceased (40d) | Foetal hydrops and bilateral pleural effusion (prenatal US). No TRECs, SCID. |
| (Saygili *et al.*, 2019) | c.1079G>T | p.G360V | Deceased (9m) | Fungal peritonitis. History of sepsis (x2). Genetic studies not available, but clinical presentation compatible with SGPL1. |

Atkinson, D. *et al.* (2017) ‘Sphingosine 1-phosphate lyase deficiency causes Charcot-Marie-Tooth neuropathy’, *Neurology*. Neurology, 88(6), pp. 533–542. doi: 10.1212/WNL.0000000000003595.

Bamborschke, D. *et al.* (2018) ‘A novel mutation in sphingosine-1-phosphate lyase causing congenital brain malformation’, *Brain & development*. Brain Dev, 40(6), pp. 480–483. doi: 10.1016/J.BRAINDEV.2018.02.008.

Janecke, A. R. *et al.* (2017) ‘Deficiency of the sphingosine-1-phosphate lyase SGPL1 is associated with congenital nephrotic syndrome and congenital adrenal calcifications’, *Human mutation*. Hum Mutat, 38(4), pp. 365–372. doi: 10.1002/HUMU.23192.

Linhares, N. D. *et al.* (2018) ‘Nephrotic syndrome and adrenal insufficiency caused by a variant in SGPL1’, *Clinical kidney journal*. Clin Kidney J, 11(4), pp. 462–467. doi: 10.1093/CKJ/SFX130.

Lovric, S. *et al.* (2017) ‘Mutations in sphingosine-1-phosphate lyase cause nephrosis with ichthyosis and adrenal insufficiency’, *The Journal of clinical investigation*. J Clin Invest, 127(3), pp. 912–928. doi: 10.1172/JCI89626.

Maharaj, A. *et al.* (2020) ‘A Sphingosine-1-Phosphate Lyase Mutation Associated With Congenital Nephrotic Syndrome and Multiple Endocrinopathy’, *Frontiers in pediatrics*. Front Pediatr, 8. doi: 10.3389/FPED.2020.00151.

Maharaj, A. *et al.* (2022) ‘Insights From Long-term Follow-up of a Girl With Adrenal Insufficiency and Sphingosine-1-Phosphate Lyase Deficiency’, *Journal of the Endocrine Society*. J Endocr Soc, 6(5). doi: 10.1210/JENDSO/BVAC020.

Mathew, G. *et al.* (2022a) ‘Infantile nephrotic syndrome, immunodeficiency and adrenal insufficiency-a rare cause: Answers’, *Pediatric nephrology (Berlin, Germany)*. Pediatr Nephrol, 37(4), pp. 817–819. doi: 10.1007/S00467-021-05377-1.

Mathew, G. *et al.* (2022b) ‘Infantile nephrotic syndrome, immunodeficiency and adrenal insufficiency-a rare cause: Questions’, *Pediatric nephrology (Berlin, Germany)*. Pediatr Nephrol, 37(4), pp. 813–815. doi: 10.1007/S00467-021-05357-5.

Pezzuti, I. L. *et al.* (2014) ‘Adrenal insufficiency in association with congenital nephrotic syndrome: a case report’, *Journal of pediatric endocrinology & metabolism : JPEM*. J Pediatr Endocrinol Metab, 27(5–6), pp. 565–567. doi: 10.1515/JPEM-2011-0296.

Prasad, R. *et al.* (2017) ‘Sphingosine-1-phosphate lyase mutations cause primary adrenal insufficiency and steroid-resistant nephrotic syndrome’, *The Journal of clinical investigation*. J Clin Invest, 127(3), pp. 942–953. doi: 10.1172/JCI90171.

Saygili, S. *et al.* (2019) ‘Persistent hypoglycemic attacks during hemodialysis sessions in an infant with congenital nephrotic syndrome: Answers’, *Pediatric nephrology (Berlin, Germany)*. Pediatr Nephrol, 34(1), pp. 77–79. doi: 10.1007/S00467-018-3982-7.

Settas, N. *et al.* (2019) ‘SGPL1 Deficiency: A Rare Cause of Primary Adrenal Insufficiency’, *The Journal of clinical endocrinology and metabolism*. J Clin Endocrinol Metab, 104(5), pp. 1484–1490. doi: 10.1210/JC.2018-02238.

Seven Menevse, T. *et al.* (2022) ‘Steroid Hormone Profiles and Molecular Diagnostic Tools in Pediatric Patients With non-CAH Primary Adrenal Insufficiency’, *The Journal of clinical endocrinology and metabolism*. J Clin Endocrinol Metab, 107(5), pp. E1924–E1931. doi: 10.1210/CLINEM/DGAC016.

Tastemel Ozturk, T. *et al.* (2023) ‘A rare cause of nephrotic syndrome-sphingosine-1-phosphate lyase (SGPL1) deficiency: 6 cases and a review of the literature’, *Pediatric nephrology (Berlin, Germany)*. Pediatr Nephrol, 38(3), pp. 711–719. doi: 10.1007/S00467-022-05656-5.

Taylor, V. A. *et al.* (2019) ‘Disarranged Sphingolipid Metabolism From Sphingosine-1-Phosphate Lyase Deficiency Leads to Congenital Nephrotic Syndrome’, *Kidney international reports*. Kidney Int Rep, 4(12), pp. 1763–1769. doi: 10.1016/J.EKIR.2019.07.018.

Tran, P. *et al.* (2023) ‘SGPL1 Deficiency: Nephrotic Syndrome with Lymphopenia’, *Journal of clinical immunology*. J Clin Immunol, 43(1), pp. 72–75. doi: 10.1007/S10875-022-01348-9.

Zhao, P. *et al.* (2020) ‘Responsiveness of sphingosine phosphate lyase insufficiency syndrome to vitamin B6 cofactor supplementation’, *Journal of inherited metabolic disease*. J Inherit Metab Dis, 43(5), pp. 1131–1142. doi: 10.1002/JIMD.12238.
